# Health related quality of life among adult hypertensive patients on treatment in Dessie City, Northeast Ethiopia

**DOI:** 10.1371/journal.pone.0268150

**Published:** 2022-09-29

**Authors:** Kidist Adamu, Amsalu Feleke, Amare Muche, Toyeb Yasin, Asnakew Molla Mekonen, Muluken Genetu Chane, Sisay Eshete, Anisa Mohammed, Abel Endawkie, Zinabu Fentaw

**Affiliations:** 1 Department of Health Systems and Management, School of Public Health, College of Medicine and Health Sciences, Wollo University, Dessie, Ethiopia; 2 Department of Health System and Policy, Institute of Public Health, College of Medicine and Health Sciences, University of Gondar, Gondar, Ethiopia; 3 Department of Epidemiology and Biostatistics, School of Public Health, College of Medicine and Health Sciences, Wollo University, Dessie, Ethiopia; 4 Department of Public Health Nutrition, School of Public Health, College of Medicine and Health Sciences, Wollo University, Dessie, Ethiopia; James Cook University Australia - Singapore Campus, SINGAPORE

## Abstract

**Introduction:**

Hypertension is one global public health emergency disease, and is one of the most critical factors for chronic diseases such as cardiovascular disease, stroke, arrhythmias, heart failure, and renal diseases. Hypertension affects quality of life of patients, however there is limited evidence on the factors that affect health related quality of life among hypertensive patients. As a result, the purpose of this study is to look into factors that affect the health-related quality of life of adult hypertensive patients.

**Methods:**

An institutional based cross-sectional study was conducted in Dessie City public health facilities from March to April 2021 with the total samples size of 376 hypertensive patients. Simple random sampling technique was employed to select study participants. World health organization health related quality of life 26 items were used to measure outcome variable. Simple linear regression analysis was done and forwarded to multivariable linear regression analysis at p-value<0.2. In multivariable linear regression analysis variables whose p-value less than 0.05 at 95% confidence interval with unstandardized β-coefficient were declared as statistically significant.

**Results:**

A total of 360 hypertensive patients were included in the study. The mean scores of physical, psychological, social and environmental domains were 42.93, SD (18.86), 51.78, SD (20.40), 57.31, SD (20.20) and 48.15, SD (16.26), respectively. Age, duration of antihypertensive treatment, low social support, physical inactivity, co-morbidity, being widow, khat chewing, and being single had a significant association with lower health related quality of life.

**Conclusion and recommendations:**

The health-related quality of life of hypertensive patients were found low in all domains. The findings indicate the necessity for health professionals, government, non-governmental organizations and community to pay more attention to patients’ quality of life, seeking changes in the therapeutic approach in general.

## Introduction

Hypertension is defined as systolic blood pressure (SBP) of 140 mmHg or higher, or diastolic blood pressure (DBP) of 90 mmHg or higher, or both. Hypertension may be a state of elevated systemic vital sign that causes marked increment of cardiovascular risk [1].

WHO has defined Quality of life (QoL) as “an individual’s perception of their position in life in the context of the culture and value systems in which they live and in relation to their goals, expectations, standards, and concerns”. Health related quality of life (HRQoL) addresses health-related domains that are impacted by elements such as physical, psychological, social, and environmental aspects. HRQoL may thus be described as a person’s perceived quality of life, indicating satisfaction in areas of life that are likely to be impacted by health condition [2]. QoL is a broad ranging concept affected in a complex way by the person’s physical health, psychological state, and level of independence, social relationships, personal beliefs, and their relationship to salient features of their environment [3, 4].

A study conducted in HRQoL among hypertensive patients showed that persons with hypertension generally scored lower in all domains of the Short form (SF)-36 instrument than did those without hypertension [2]. Another population-based study showed that hypertension was associated with increased risk of having a worse HRQoL, representing 14 or more unhealthy days per month compared with normotensive individuals, an indication of lower quality of life of individuals with hypertension [5].

Factors like longer duration hypertension, low level of education, presence of hypertension related symptoms, being divorced, uncontrolled blood pressure status, age, adherence to treatment, presence of co-morbidity, complication, being farmer, being female, more than one symptom count, having stroke, visual impairment [5–8], have significant association with HRQoL of Hypertension patients. Smoking and alcohol consumption has a negative relation with good HRQoL for others diseases, Whereas studies conducted in Ethiopia have not addressed the life style factors affecting the HRQoL of hypertension patients [6, 7].

Low health related quality of life has profound negative effect on health institutions and patients. Low level of health-related quality of life in health institutions increases patients flow, professional demand and demand of infrastructure. Low HRQoL at the patients’ level also increases hospitalization, decreases patients’ income, social relationship, increases psychological problem, and physical disability [9].

Despite, Ethiopian government efforts to reduce the incidence of hypertension and related complications in house to house, market days screening for early diagnosis, prevention and treatment accessibility, but the physical, psychological, social, and environmental quality of life of hypertensive patients living in communities has not been adequately addressed. Evidences show that hypertensive patients in Ethiopia had low quality of life as compared with sub-Saharan and the global communities [10].

Generally, there are limited studies regarding health-related quality of life and its associated factors on hypertension patients. This study considers the life style factors and perceived social support on the HRQoL of hypertension patients. Therefore, the main aim of this study is to assess health related quality of life and factors associated with health-related quality of life of hypertensive patients with respect to Physical, Psychological, Social and Environmental domains.

## Methods and materials

### Settings and design

Institution based cross-sectional study was conducted among adult hypertensive patients on treatment at Public Health Facilities of Dessie City Administration from March to April 2021. The city is located 401 kilometers away from Addis Ababa to the Northeast. There are 8 governmental Health Facilities, which were providing antihypertensive treatment for the total of 1633 adults. Hypertension treatment is given on a monthly basis through checking their blood pressure and dispensing appropriate drugs.

### Participants, sample size determination and sampling procedures

Hypertensive patients were age 18 years and above, and on antihypertensive medication for six months or more were eligible for this study. While hypertensive patients with critical illness, persons with disability (unable to communicate) and pregnant women were excluded from this study. The number of participants were computed using single population mean formula, then the largest sample size was obtained from Role Physical domain (RP) with standard deviation 47.16 at 95% confidence level and an error of 5 [7]. So, final maximum sample size was 376 hypertensive patients. The participant of this study was recruited using simple random sampling method from their appointment charts through computer generating random number.

### Data collection method and tools

The data were collected using a structured interview administered questionnaire, which was prepared to address all the important variables with supportive document review using patients’ chart after obtaining an informed consent while exiting from their clinical care. The data collection tool contains sociodemographic, clinical profile of the patient and social and behavioral practice of hypertensive patients. The questionnaires were adapted from different literatures of similar studies [6, 7]. Health related quality of life questionnaire was adopted from the WHOQoL-BREF- 26 items validated cheek list that consist four domains [11].These are physical health (7 items), psychological health (6 items), social relationships (3 items), and environmental health (8 items), and two items not included in any of the domains are overall perception of QoL and general health perception. Each of these items was scored from 1 to 5 on a response scale, which is agreed as a five point Likert scale [12]. This validated WHOQoL-BREF-26 items, was obtained from WHO website with Amharic version, it was directly used for assessing the health-related quality of life of hypertensive patients. The perceived social support related questionnaire was adopted from multidimensional scale of perceived Social support (MSPSS)-12 item check list [13], which was validated with a similar setting [14].

### Data quality control

The questionnaires were developed in English then translated to Amharic language and also retranslated into English language by Amharic and English language experts, respectively. The data collectors were selected in the governmental health institutions, who were working other than the selected study areas. For the verification of data quality, pre-test was conducted in Kombolcha Town 03 Health Center, two weeks prior to the actual data collection period for 19 clients to check the clarity of the questions and necessary amendments was done on the questionnaire. The reliability of the tools was also tested for internal consistency using Cronbach’s α.

### Data processing and analysis

Data were checked for completeness, then entered in to Epi Data version 3.1 and exported to STATA/SE14.0 for further analysis. Also, the data were coded, cleaned and explored to identify missing values, outliers and inconsistency through tabulation and graphical display. Dummy variables (for k categories, k-1 dummy variable) were created for categorical variables.

Reverse coding was done on negative items in physical and psychological domain of the outcome variables and then computing a raw score of each domain. The scores of each domain were transformed to 0–100 transformed scores using WHOQoL-BREF scoring guideline [12]. The perceived social support was also transformed in three categories using tertile of their computed raw scores. The lowest, middle and highest tertiles were recoded as low, moderate and high social supports, respectively [13].

The data were presented using texts, tables and graph. Continuous variables including the outcome of the study were described in terms of mean with their standard deviation and median with their interquartile range. Categorical characteristics were described in terms of frequencies and percentages.

All necessary assumptions of linear regression were checked and fulfilled. Then linear regression analysis was performed to identify factors associated with health-related quality of life with respect to physical health domain, psychological health domain, social health domain and environmental health domain. Simple linear regression analysis was done then variables with p-value <0.2 were included into multiple linear regression analysis [7].

In a forward multivariable linear regression analysis variables whose p-value less than 0.05 at 95% confidence interval with unstandardized β-coefficient of the predictor variables were declared as statistically significant.

### Ethical issues

Ethical clearance was obtained from Ethical Review Committee of Wollo University College of Medicine and Health Sciences. The respondents were informed about the purpose of the study, and written consent was obtained from each participant. The data were collected in accordance with Helsinki declaration. The respondents’ right to refuse or withdraw from participation of the interview is fully maintained and the information provided by each respondent was kept strictly confidential.

## Result

### Socio-demographic characteristics

From a total of 376 study participants, 360 hypertensive patients participated in the study, which gave a response rate of 95.7%. Among the total respondents, 207(57.5%) were females. The mean age of the respondents was 55.9 with a standard deviation (SD) of 11.56, at a minimum and a maximum of 20 and 88 years old, respectively. Of the participants, 160(44.4%) were unable to read and write and, with respect to occupational status, 92 (25.6%) were house wives. The median monthly income of participants was 2750 with an interquartile range (IQR) of 2554 Ethiopian birr. Regarding marital status and residence, 244(67.8%) and 258(71.7%) were married and residing in urban areas, respectively [Table 1].

**Table 1:** Characteristics of hypertensive patients on treatment attending public health facilities in Dessie City Administration, Dessie, Northeast Ethiopia 2021 (360).

Variables	Categories	Frequency(count)	Percent (%)
Sex	Male	153	42.5
	Female	207	57.5
Occupation	Government employed	53	14.7
	Private employed	31	8.6
	Merchant	39	10.8
	Farmer	66	18.3
	Unemployed	26	7.2
	Daily laborer	36	10.0
	House wife	92	25.6
	Pension	17	4.7
Education	Cannot read and write	160	44.4
	Can read and write	66	18.3
	Primary school	38	10.6
	Secondary and preparatory	59	16.4
	College and above	37	10.3
Marital status	Single	22	6.1
	Married	244	67.8
	Widowed	63	17.5
	Separated	31	8.6
Residence	Urban	258	71.7
	Rural	102	28.3

### Clinical, life style and psychosocial characteristics of study participants

The median duration of hypertension since diagnosis was 7 years with an IQR of 7.5years. The median duration of anti-hypertensive treatment was 6 years with an IQR of 7 years. Regarding hypertension related complications 130(36.1%) of participants developed complication. Out of the total participants 131(36.4%) had co-morbid diseases. The mean body mass index of participants was 23.61, SD (3.46) Kg/m2. In this study 303(84.2%) of participants had one or more symptoms and 235(65.3%) of participants were taking two or more drugs. From total participants 332(92.22%) had good adherence and 232(64.44%) of participant had controlled blood pressure status. From the total participants 14(3.9%), 21(5.8%), and 34(9.4%) were smokers, alcohol consumers and khat chewers, respectively. Only 108(30%) of study participants did physical exercise, and 309 (85.8%) of participants restricted their salt consumption. Regarding to perceived social support 85(23.6%) participants had low social support, 143(39.7%) had moderate social support and 132(36.7%) had high social support [**Table 2**].

**Table 2:** Clinical and life style factors of hypertension patients on treatment attending public health facilities in Dessie City Administration, Dessie, Northeast Ethiopia 2021.

Variable	Categories	Frequency	Percent
Number of Drugs	Only one	125	34.7
	Two or more drug	235	65.3
Number of Symptoms	No symptom	57	15.8
	One or more symptom	303	84.2
Co-morbidity	No	229	63.6
	Yes	131	36.4
Complications	No	230	63.9
	Yes	130	36.1
Adherence	Good	332	92.2
	Poor	28	7.8
Blood Pressure Status	Controlled	232	64.4
	Uncontrolled	128	35.6
Smoking	Yes	14	3.9
	No	346	96.1
Alcohol	Yes	21	5.8
	No	339	94.2
Physical Exercise	Yes	108	30.0
	No	252	70.0
Khat Chewing	Yes	34	9.4
	No	326	90.6
Salt Restriction	Yes	309	85.8
	No	51	14.2
Perceived Social Support	Low	85	23.6
	Moderate	143	39.7
	High	132	36.7

### HRQoL among adult hypertensive patients

The four domains internal reliability with Cronbach’s α were: Physical α = 0.84, Psychological α = 0.85, Social α = 0.68 and Environmental α = 0.75. The mean score of physical, psychological, social and environmental domain were 42.93,SD(18.86), 51.78,SD(20.40), 57.31,SD(20.20) and 48.15,SD(16.26), respectively [**Table 3**].

**Table 3:** Health related quality of life domain score of hypertension patients on treatment attending public health facilities in Dessie City Administration, Dessie, Northeast Ethiopia 2021.

HRQoL domains	Mean (SD)	95%CI
Physical Domain	42.93(18.86)	40.98–44.89
Psychological Domain	51.78(20.40)	49.66–53.89
Social Domain	57.31(20.20)	55.22–59.4
Environmental Domain	48.15(16.26)	46.46–49.83

### Health status satisfaction and self-rating of HRQOL of hypertensive patients

From the total study participants 142 (39.4%) were poor in their self-rating quality of life whereas out of the total study participants 102 (28.3) were dissatisfied in their general health perception [**Table 4**].

**Table 4:** Health status satisfaction and self-rating of health related quality of life of hypertension patients on treatment attending public health facilities in Dessie City Administration, Dessie, Northeast Ethiopia 2021.

Self- reported quality of life	Frequency	Percent	Self-reported health satisfaction	Frequency	Percent
Very Poor	38	10.6	Very Dissatisfied	63	17.5
Poor	142	39.4	Dissatisfied	102	28.3
Neither Poor nor Good	163	45.3	Neither Dissatisfied nor Satisfied	168	46.7
Good	12	3.3	Satisfied	20	5.6
Very Good	5	1.4	Very Satisfied	7	1.9

### Factors associated with HRQoL of adult hypertensive patients on treatment

#### Factors associated with physical domain of HRQoL of adult hypertensive patients on treatment

The variables, which were entered into simple linear regression analysis and passed to multivariable linear regression analysis with p- value <0.2, were age, widowed, self-employed, monthly income, duration of hypertension(HTN) since diagnosis, duration of HTN since treatment, smoking, khat chewing, BMI, low social support, complication, comorbidity, number of symptoms and uncontrolled BP status [**Table 5**].

**Table 5:** Multivariable linear regression model on factors associated with physical domain of HRQoL among adult hypertensive patients on treatment at public health facilities in Dessie City Administration, Northeast Ethiopia, 2021 (n = 360).

Variables	Bi-Variable regression	Multiple Linear Regression		
	β(95% CI)	β(95% CI)	Standard error	P-Values
Age	-0.27(-0.44,-0.10)	-0.26(-0.40,- 0.11)	0.07	0.00
Duration of HTN since Rx	-0.58(-0.93, -0.23)	-0.94(-1.82,-0.07)	0.45	0.03
Social support				
Low High (reference)	-19.30(-23.45,15.15)	-16.58(-20.55,-12.61)	2.02	0.00
Complication No (reference)	-4.94(-8.98, -0.89)			
Comorbidity No (reference)	-9.86(-13.79, -5.92)	-7.17(-10.59,-3.74)	1.74	0.00

Non-significant variable in multiple linear regression: marital status, occupation, Income, duration of hypertension since diagnosis, Smoking, Khat Chewing, BMI, Complication, Number of symptoms and Blood pressure control status.

Then multivariable linear regression analysis was done. Variables like age, duration of treatment, low social support, and co-morbidity were declared as having significant association with physical health related quality of life of hypertension patients [**Table 5**].

As age increases by one-year patients physical health related quality of life decreased on average by 0.26 unit keeping the effect of other variables constant [-0.26,95%CI (-0.40 to -0.11)].

As duration of anti- hypertension treatment increases by one year patients physical health related quality of life decreased by 0.94 unit keeping the effect of other variables constant [-0.94, 95%(-1.82 to -0.07)].

Patients who had low social support were 16.58 lower physical health related quality of life as compared to patients who had high social support keeping the effect of other variables constant [-16.58 95%CI(-20.55 to -12.61)].

Patients who had co-morbid illness were-7.16 lower physical health related quality of life as compared to those patients who did not have co-morbid illness keeping the effect of other variables constant [-7.16,95% CI(-10.59 to -3.74)] [**Table 5**].

#### Factors associated with psychological domain of HRQoL of adult hypertensive patients on treatment

The variables which were entered into simple linear regression analysis and passed to multivariable linear regression analysis with p- value <0.2 were sex, informal education, marital status, self- employed, average monthly income, duration of hypertension since diagnosis, duration of hypertension treatment, BMI, smoking, low social support, salt restriction, complication, one or more symptom, and uncontrolled BP status.

Then multivariable linear regression analysis was done. Variables like being widowed, khat chewing, and low social support were declared as statistically significant association with psychological health related quality of life of hypertension patients.

Being widowed was on average 5.16 lower psychological health related quality of life as compared with married keeping effect of other variables constant [-5.16, 95% CI (- 10.17 to -0.15)].

Khat chewer hypertension patients were7.63 lower psychological health related quality of life as compared to Non- chewers keeping the effect of other variable constant [-7.63,95%CI(-13.99 to -1.26)].

Hypertension patients with low social support were 19.59 lower psychological health related quality of life as compared with high social support keeping the effect of other variables constant [-19.59,95%CI(-23.99 to -15.19)] [**Table 6**].

**Table 6:** Multivariable linear regression model on factors associated with psychological domain of HRQoL among adult hypertensive patients on treatment at public health facilities in Dessie City Administration, Northeast Ethiopia, 2021 (n = 360).

Variable	Bi-variable regression		Multiple linear regression		
	β(95%CI)		β(95%CI)	Standard error	P-values
Marital status					
Single	-6.65(-15.46, 2.16)				
Widowed	-7.67(-13.19, -2.16)		-5.16(-10.17,-0.15)	2.55	0.04
Khat chewing No (reference)	-10.78(-17.93, -3.62)		-7.62(-13.99,-1.26)	3.24	0.01
Low social support High (reference)	-22.75(-27.14, -18.36)		-19.59(-23.99,-15.19)	2.24	0.00

Non-significant variable in multiple linear regression: sex, Educational status, Occupational status, average monthly income, Duration of HTN since diagnosis, Duration of HTN since treatment, BMI, Smoking, salt restriction, salt restriction, complication, number of symptoms and blood pressure control status.

#### Factors associated with social domain of HRQoL of adult hypertensive patients on treatmen

The variables which were entered into simple linear regression analysis and passed to multivariable linear regression analysis with p- value <0.2 were age, informal education, single, self-employed, unemployed, income, duration of HTN since diagnosis, duration of anti-hypertension treatment, khat, salt restriction, BMI, low social support, complication, co-morbidity, one or more symptoms and uncontrolled BP status.

Then multivariable linear regression analysis was done. Variables like being single and low social support were declared as statistically significant association with social health related quality of life of hypertension patients.

Being single was on average 12.42 lower social health related quality of life as compared with married keeping the effect of other variables constant [-12.42,95%CI (-20.57 to -4.27)].

Patients who had low social support was on average 13.81 lower social health related quality of life as compared with patients who had high social support [-13.81,95%CI (-18.49 to -9.14)] [**Table 7**]

**Table 7:** Multivariable linear regression model on factors associated with social domain of HRQoL among adult hypertensive patients on treatment at public health facilities in Dessie City Administration, Northeast Ethiopia, 2021 (n = 360).

Variable	Bi-variable linear regression		Multiple linear regression		
	β(95%CI)		β(95% CI)	Standard error	p-value
Marital status					
Single Married (reference)	-14.86(-23.48, -6.25)		-12.42(-20.57,-4.27)	4.14	0.00
Social support					
Low High (reference)	-17.24(-21.84, -12.64)		-13.81(-18.49,-9.14)	2.38	0.00

Non-significant variable in multiple linear regression: age, educational status, occupation, average monthly income, duration of hypertension since diagnosis, duration of diagnosis since treatment, Khat Chewing, salt restriction, BMI, complication, comorbidity, number of symptoms and blood pressure control status.

#### Factors associated with environmental domain of HRQoL of adult hypertensive patients on treatment life

The variables which were entered into simple linear regression analysis and passed to multivariable linear regression analysis with p- value <0.2 were sex, informal education, self-employed, duration of HTN since diagnosis, duration of anti-hypertension treatment, physical exercise, salt restriction, BMI, low social support, complication, co-morbidity, one or more symptoms and uncontrolled BP status.

Then multivariable linear regression analysis was done. Variables like physical exercise, low social support, and co-morbidity were declared as statistically significant association with environmental health related quality of life of hypertension patients.

Patients who did not perform physical exercise was on average 8.18 lower environmental health related quality of life as compared with those who did physical exercise [-8.18,95%CI(-11.33 to -5.04)].

Patients who had low social support was on average 15.47 lower environmental health related quality of life as compared with patients who had high social support keeping the effect of other variables constant [-15.47,95%CI(-18.94 to -11.99)].

Patients who had co-morbid disease was on average 5.25 lower environmental health related quality of life as compared with those without co-morbid disease keeping the effect of other variables constant [-5.25,95%CI (-8.24 to -2.27)] [**Table 8**].

**Table 8:** Multivariable linear regression model on factors associated with environmental domain of HRQoL among adult hypertensive patients on treatment at public health facilities in Dessie City Administration, Northeast Ethiopia, 2021 (n = 360).

Variable	Bi–variable linear regression		Multiple linear regression		
	β(95%CI)		β(95%CI)	Standard error	p-value
Physical exercise Yes (reference)	-7.35(-10.96, -3.75)		-8.18(-11.33,-5.04)	1.60	0.00
Social support					
Low High (reference)	-17.38(-20.93, -13.84)		-15.47(-18.94,-11.99)	1.77	0.00
Comorbidity No (reference)	-8.23(-11.63, -4.82)		-5.25(-8.24,-2.27)	1.52	0.00

Non-significant variable in multiple linear regression: sex, occupational status, duration of hypertension since diagnosis, duration of treatment since treatment, salt restriction, BMI, complication, number of symptoms and blood pressure control status.

## Discussion

The mean score of all domains of the health-related quality of life of hypertensive patients was low. In this study, the mean score of all domains of health-related quality of life of hypertensive patient was lower than a study conducted in Vietnam [8], Iran [15], Brazil [16] and Nigeria [17]. But, psychological domain mean score of this study was found similar with the study conducted on Vietnam. This variation for being lower than the study’s conducted in other countries may be due to high socio-economic differences across each country [18], the level of satisfaction with the infrastructure and health care service is higher in the countries with high quality of life among hypertensive patients [8, 19] than Ethiopia [20].

The clinical characteristics of the patients also different like patients with co-morbidity and complication were lower among these studies [8, 17]. The other important contributing factors that claim to have an influence for this variation is also level of literacy. Those countries with higher level of literacy have a good quality of life. According to UNICEF report, the national level of literacy of the above countries are higher than the literacy rate in Ethiopia [21]. The other factors that lead to this level of difference is may be due to the health care system discrepancy across each country. The health care delivery system in Brazil, Iran and Vietnam having a package of non-drug therapy, frequent visit of the health facilities, as well as higher ratio of physician to patients than Ethiopia [22–24].

Whereas, the finding of the current study was higher than the study conducted in Nigeria Pieta and Hill Crist hospitals. This may be due to the type of transformed scores and measurement variation. The study was using the same tools but, the study conducted in two hospitals were not transformed its raw score into zero to hundred scores [25].

Aging is negatively associated with patients physical HRQoL. This study finding was consistent with studies conducted in America, China, Nepal, Vietnam, Sweden and Poland [16, 17, 26–29] Biologically, aging is characterized by a gradual, and lifelong accumulation of molecular and cellular damage that subsequently leads to a decrease in physiological functions increased vulnerability to diseases and a general decline in the capacity of the individual [18] As people age increases, there is a decline in vision and hearing, waned musculoskeletal strength and function and compromised immune function. With increasing age, people are more likely to experience multi-morbidity, which has a significant impact on HRQoL the impact being greater than the additive effect of individual conditions [19]. The clinical implication of this finding is when the age of individual hypertensive patients increases, they prone to physical disability and mental disorder. Due to this reason, they need close follow up, physical rehabilitation therapy, and continuous psychological support.

Marital status had a significant association with the social domain health related quality of life of hypertensive patients. Those who were single were more likely to have a lower social and environmental HRQoL compared to married ones. This is consistent with the study conducted in Sweden, Brazil and Oman [29–31]. The reason for this is mental problems, feeling of loneliness related to being single are more common, and people may get socially isolated over time [32]. This implies that these patients need close follow-up from physicians and the community.

Those who were widowed were more likely to have lower psychological domain of HRQoL compared to married hypertensive patients. This is consistent with the study conducted in Sweden [29]. The possible explanation for the lower quality of life among widowed may be due to increased psychological problems, feeling lonely, anxiety, depression and lack of confidence in the community [33]. The finding has given a clue to give emphasis on psychological support of these patients in clinical area and in community level.

This study showed that patients with co-morbidities had a lower score in the physical and environmental domains of HRQoL than patients without co-morbidities. This finding is consistent with the study conducted in Mekelle and Jimma [6, 7] The reason for this is co-morbidities are associated with greater health care needs, greater likelihood of disability, increased cost of care, higher likelihood of financial burden, and resulting socio-economic disadvantage [34]. All of these can be associated with impaired quality of life. The lower QoL among hypertensive patients with co-morbidities supports the importance of early diagnosis and effective treatment of chronic conditions to preserve high QoL in these patients.

This study showed that patients who had low social support were more likely to have lower physical, psychological, social and environmental domains of HRQoL compared to those patients who had high social support. This might be due to the fact that patients with low social support had worse perceived health, more pain, worse physical functioning, greater difficulties with daily activities, more health related–distress, worse cognitive functioning, wore perceived quality of life, wore physical and emotional health than in the preceding month, and in overall terms worse HRQoL than participants with a high level of social support [35].

The environmental and psychological HRQoL scores of khat chewers in this study were lower than non-khat chewers. The HRQoL of khat chewers were low compared with non-chewers. This might be due to the fact that khat is sometimes associated with mental health problems, such as depression, paranoia, hallucination, manic behavior, hyperactivity, and some other mental disorders [36, 37]. Moreover khat chewing had been associated with gastrointestinal problems, such as mouth ulcers, inflammation of the esophagus and stomach, gum disease, jaw problems, constipation and affect socio-economic status [30]. This implies that the need to increase law enforcement efforts and community-based interventions focusing on social networking to fight with khat use habit.

Longer duration of anti-hypertensive treatment was associated with lower physical HRQoL. The long-term use of antihypertensive agents causes adverse effects and tolerances [38]. Some kind of antihypertensive agents have an effect on the metabolic aspects, particularly like the worsening of the serum lipids and lowering of the glucose tolerance [31].

Physical activity had a significant association with environmental domains of HRQoL. Those who did not perform physical exercise were more likely to have a lower HRQoL compared to those who did physical exercise. This is in line with the study conducted in Vietnam [8]. This might be due to the fact that people who are inactive tend to have higher resting heart rates. The higher the heart rate, the harder the heart must work with each contraction and the stronger the force on the arteries [39]. Regular exercise tends to reduce the risk of complications of hypertension and improves the feeling of well-being. The improvements in HRQoL associated with physical activity might be considered to be ‘process benefits’ that arise from engagement in physical activity. These might occur due to the process of participating (increased social interactions resulting from group participation or time spent outdoors), improved self-esteem (positive perceptions of competences and the physical-self) or biologic mechanisms (increased endorphin levels as a result of Physical activity) [40, 41].

### Limitation of the study

Because this was a cross-sectional study, it had the same limitations as other cross-sectional studies, because the outcome and predictors variables are measured at the same time, it is relatively difficult to establish causal relationships from a cross-sectional study.

## Conclusions

Hypertension has an effect on the patient’s HRQoL. All the scores of the domain of health-related quality of life of hypertensive patients are low.

Old age, increased duration of anti-hypertensive treatment, having low social support, and co-morbidities were inversely associated with physical domain of health-related quality of life.

Being widowed, having low social support, and khat chewing were negatively associated with psychological domain of health-related quality of life.

Being single and having low social support were also negatively associated with social health related quality of life.

Being physically inactive, having low social support, and co-morbidity were negatively associated with environmental domains of health related quality of life.

## Supporting information

S1 Dataset (XLS)
